# Predictors of suicidal ideation among female residents in a community impacted by multiple natural disasters.

**DOI:** 10.1192/j.eurpsy.2023.2012

**Published:** 2023-07-19

**Authors:** B. Agyapong, R. Shalaby, E. Eboreime, Y. W., V. Agyapong

**Affiliations:** Department of Psychiatry, University of Alberta, Edmonton, Canada

## Abstract

**Introduction:**

Suicidal ideation and thoughts of self-harm continue to be challenging public health problems. It’s presently unknown what the prevalence and correlates of suicidal thoughts and self-harm are in female residents of Fort McMurray, a city that has endured wildfires, flooding, and the COVID-19 pandemic in the last five years.

**Objectives:**

This study aimed to determine the prevalence and correlates of suicidal ideation and thoughts of self-harm among female residents of Fort McMurray.

**Methods:**

A cross-sectional study using an online survey questionnaire was used to collect sociodemographic and clinical information from the resident of Fort McMurray between April 24 to June 2 2021. Suicidal ideation and thoughts of self-harm among females were assessed using the ninth question of the Patient Health Questionnaire-9, a validated screening tool used to assess depression symptoms. Likely Generalized Anxiety Disorder (GAD), Major Depressive Disorder (MDD), Post-Traumatic Stress Disorder (PTSD) and low resilience were measured using standardized rating scales. Data were analyzed with SPSS version 25 using Chi-Square tests and multivariate logistic regression analysis.

**Results:**

Among Fort McMurray residents, 249 accessed the online survey, while 186 ultimately completed it, yielding a survey completion rate of 74.7%. Of these, 159 (85%) were females. After controlling for other variables in the regression model, respondents who expressed a desire to receive mental health counselling were more than seven times more likely to report suicidal ideation and thoughts of self-harm compared to the respondents who didn’t desire to receive mental health counselling (OR: 7.29; 95% CI: 1.19 – 44.58). Similarly, respondents who reported having abused alcohol in the past year were nearly four times more likely to report suicidal ideation or thoughts of self-harm compared to the respondents who said they had not abused alcohol in the past year (OR: 3.91; 95% CI: 1.05 – 14.57).

**Conclusions:**

High prevalence of suicidal thoughts and thoughts of self-harm were reported among female residents of Fort McMurray. Timely access to adequate mental health support should be offered to female residents of communities impacted by multiple natural disasters, particularly residents who self-report alcohol abuse or desire to receive mental health counselling

**Disclosure of Interest:**

None Declared

